# Left-Sided Transudative Chylothorax With Concomitant Chylous Ascites in the Setting of Liver Cirrhosis

**DOI:** 10.7759/cureus.33866

**Published:** 2023-01-17

**Authors:** Janet Gukasyan, Alexander T Phan, Janie Hu, Shuhab Zaher, Sarkis Arabian, Mufadda Hasan, Dan Vo

**Affiliations:** 1 School of Medicine, California University of Science and Medicine, Colton, USA; 2 Internal Medicine, Arrowhead Regional Medical Center, Colton, USA; 3 Pulmonary and Critical Care, Arrowhead Regional Medical Center, Colton, USA; 4 Pulmonary and Critical Care Medicine, Arrowhead Regional Medical Center, Colton, USA

**Keywords:** pleural disease, hepatology, pulmonary rehabilitation and medicine, pulmonary medicine, alcohol related cirrhosis, left sided pleural effusion, chylothorax

## Abstract

Most chylothoraces are caused by trauma and malignancy, and pleural fluid analysis typically demonstrates an exudative effusion. Transudative chylothorax is a rare manifestation and has only been cited in case reports in the current literature. Here, we present the case of a 59-year-old male with a history of liver cirrhosis secondary to alcohol abuse, chronic kidney disease stage 3a, and hypertension who presented with a left-sided pleural effusion and abdominal ascites. A thoracentesis and abdominal paracentesis were performed, and fluid analyses demonstrated a transudative chylothorax with concomitant chylous ascites. In this review, we aim to highlight a rare case of transudative chylothorax and discuss the pathogenesis and management of this condition.

## Introduction

Chyle is a fluid with a milky appearance that is found in lymphatic vessels, consisting of cholesterol, triglycerides, chylomicrons, fat-soluble vitamins, and lymph, which contains immunoglobulins, digestive products, and leukocytes [1]. A chylothorax is the accumulation of chyle in the pleural cavity and may present as a pleural effusion [2]. The mechanism behind chylothorax development involves thoracic duct obstruction or injury or transdiaphragmatic flow of peritoneal fluid [1-2]. The causes of chylothorax are subcategorized into traumatic (e.g., direct tissue damage) or nontraumatic (e.g., malignancy, sarcoidosis, amyloidosis, and superior vena cava thrombosis), with traumatic etiologies being the most common [1]. Of the non-traumatic causes, malignancy is estimated to cause up to 46% of all cases, with thoracic duct obstruction being the most common mechanism [2].

Clinically, patients with chylothorax present with typical symptoms of pleural effusions, including dyspnea, chest pain, and cough [1]. It is uncommon for patients to present with signs of pleurisy, as the chyle does not irritate the pleural lining [2]. The loss of immunoglobulins, other proteins, and T-lymphocytes via lymphatic fluid extravasation into the thoracic cavity may cause patients to become immunosuppressed. Additionally, significant loss of chyle may produce symptoms of hypovolemia and warrant fluid resuscitation [1]. Chylothoraces are typically unilateral and most commonly appear on the right hemithorax, as a result of the location of the thoracic duct [1-2].

The gold standard for the diagnosis of a chylothorax is a pleural fluid triglyceride >110 mg/dL; however, if pleural fluid triglycerides are <110 mg/dL and clinical suspicion for a chylothorax remains high, the presence of chylomicrons may confirm a diagnosis of a chylothorax [2]. Pleural fluid analyses of chylothoraces have previously demonstrated that the large majority are exudative in nature [2]. A transudative chylous effusion is exceedingly rare, and some small reports have cited cirrhosis and nephrosis as the most common etiologies [1]. Here, we present a case of transudative chylothorax in a cirrhotic patient with concomitant chylous ascites.

## Case presentation

A 59-year-old male with a history of hepatic cirrhosis secondary to alcohol abuse, chronic kidney disease stage 3a, and hypertension presented to the emergency department with a three-month history of progressive dyspnea on exertion, orthopnea, an enlarging abdomen, and leg swelling. Of note, he was recently diagnosed with hepatic cirrhosis six months prior during a previous hospitalization where a paracentesis demonstrated portal hypertension; ascitic fluid triglyceride levels were not obtained at that time. The patient denied any history of spontaneous bacterial peritonitis and denied requiring frequent large-volume abdominal paracentesis. Regarding his chronic kidney disease, prior urine studies did not demonstrate nephrotic range proteinuria. He denied fevers, chills, nausea, vomiting, abdominal pain, melena, hematochezia, or hemoptysis. His social history was significant for alcohol abuse, quantified at six alcoholic beverages daily, with his last alcohol consumption occurring six months prior to admission. His home medications included oral furosemide 40 mg daily and oral spironolactone 100 mg daily, with which he had been compliant.

**Figure 1:**
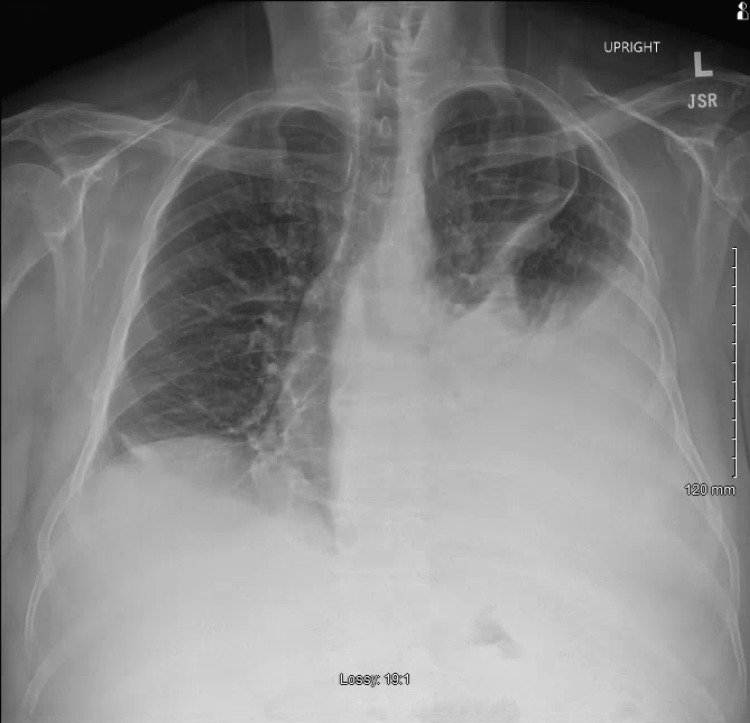
Anterior-posterior chest radiograph demonstrating a large left-sided pleural effusion and small right-sided pleural effusion.

**Figure 2:**
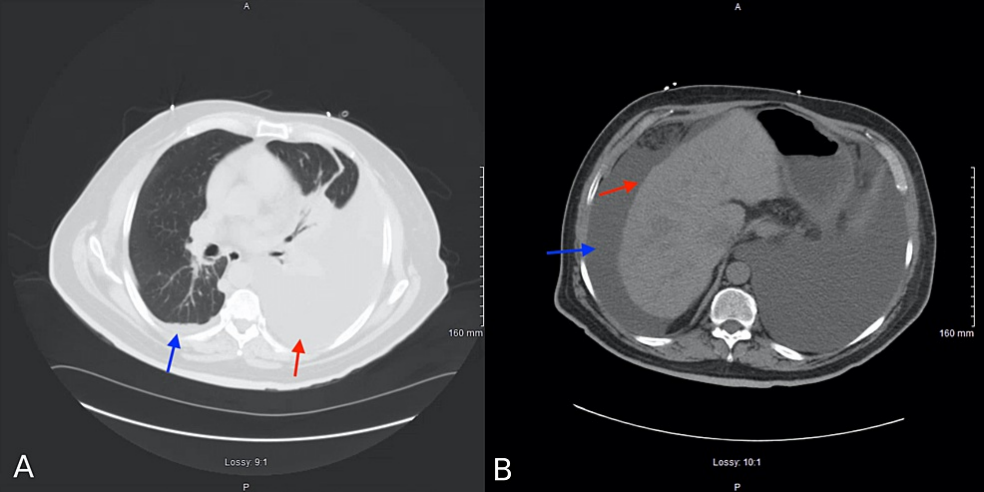
(a) Axial section of a computed tomography of the chest demonstrating a large left-sided pleural effusion with atelectasis (red arrow) and small right-sided pleural effusion (blue arrow). (b) Axial section of a computed tomography of the abdomen demonstrating nodular liver contour (red arrow) and large ascites (blue arrow).

On admission, his vital signs included a blood pressure of 95/64 mmHg, a pulse rate of 71, a temperature of 97.7 °F, a respiratory rate of 14, and an oxygen saturation of 98% on 4 L of low-flow nasal cannula oxygen supplementation. The patient’s initial laboratory findings are listed in Table 1, demonstrating leukopenia, macrocytic anemia, thrombocytopenia, mild hyperbilirubinemia, coagulopathy, impaired renal function, and hypoalbuminemia. On physical exam, the patient was noted to have scleral icterus, 2+ pitting edema bilaterally of the lower extremities extending through the mid-shin, abdominal distension with a positive fluid wave, and diminished breath sounds over the lower half of the left hemithorax. A chest X-ray obtained revealed a large left-sided pleural effusion (Figure 1). Computed tomography of the chest, abdomen, and pelvis demonstrated a large left-sided pleural effusion with atelectasis, a small right-sided pleural effusion, nodular liver contour, and large abdominal ascites (Figure 2A-2B). There were no masses or issues with the diaphragm or lymph nodes noted on CT imaging. His MELD-Na score was calculated to be 20 and Child’s Pugh classification was graded at Stage B.

**Table 1:** Initial laboratory studies demonstrating leukopenia, macrocytic anemia, thrombocytopenia, mild hyperbilirubinemia, coagulopathy, impaired renal function, and hypoalbuminemia. BUN: blood urea nitrogen, μL: microliter, g: gram, U: unit, dL: deciliter, fL: femtoliter, mEq: milliequivalent, mmol: millimole, AST: aspartate aminotransferase, ALT: alanine aminotransferase.

Laboratory test	Reference values	Measured values
White blood cells (cells/μL)	4,300–11,100	3,900
Hemoglobin (g/dL)	11.5–15.5	9.5
Hematocrit (%)	36–46	29
Platelet (cells/μL)	120,000–360,000	99,000
Mean corpuscular volume (fL)	80–100	104
Total bilirubin (mg/dL)	0–1.2	1.3
INR	<1.1	1.47
Sodium (mEq/L)	135–148	135
Potassium (mEq/L)	3.5–5.5	3.9
Chloride (mEq/L)	98–110	98
Bicarbonate (mmol/L)	24–34	27
BUN (mg/dL)	8–20	29
Creatinine (mg/dL)	0.5–1.5	1.83
Albumin (g/dL)	3.5–4.9	2.4
AST (U/L)	5–40	46
ALT (U/L)	5–40	19

A bedside ultrasound-guided paracentesis removed 5 L of straw-colored fluid, and 25 g of intravenous (IV) albumin was administered. Following this, an ultrasound-guided, left-sided thoracentesis was performed, yielding 1 L of pink and milky fluid. The peritoneal fluid analysis demonstrated chylous ascites with a serum-ascites albumin gradient of 2.2 (Table 2). Pleural fluid analysis showed a transudative chylous effusion by Light's criteria and cholesterol criteria (Table 2). The patient was thus diagnosed with a transudative chylothorax with concomitant chylous ascites.

**Table 2:** The patient's peritoneal fluid analysis, pleural fluid analysis, serum LDH, and serum total protein. LDH: lactate dehydrogenase, U: unit, L: liter, μL: microliter, g: gram, mg: milligram, dL: deciliter.

Laboratory test	Reference values	Measured values
Peritoneal albumin (g/dL)	3.5–4.8	4.6
Peritoneal white blood cells (cells/μL)	<500	226
Peritoneal lymphocytes (%)	25–33	85
Peritoneal triglycerides (mg/dL)	0	329
Pleural pH	7.0–7.5	7.42
Pleural total protein (g/dL)	1–2	2.6
Pleural LDH (U/L)	n/a	100
Pleural cholesterol (mg/dL)	0	39
Pleural triglycerides (mg/dL)	0	593
Serum LDH (U/L)	120–230	262
Serum total protein (g/dL)	6–8	6.8

Urine electrolytes were obtained, demonstrating urine sodium of <10. Due to concern for hepatorenal syndrome, the patient underwent an albumin challenge with IV albumin 1 g/kg body weight. Since his creatinine did not improve after two days, the patient was started on treatment for suspected hepatorenal syndrome with IV albumin 50 g daily, IV octreotide 100 mcg three times daily, and oral midodrine 7.5 mg three times daily. The hepatology specialist was consulted and recommended transfer to a liver transplant center. In the following 16 days, while awaiting transfer to a liver transplant center, the patient underwent two more paracenteses (4 L and 5 L removed each time), which continued to demonstrate chylous ascites with peritoneal triglycerides of 152 and 180 mg/dL. Alpha-fetoprotein levels in the ascitic fluid were not measured. On hospital day 17, the patient was transferred to another institution for liver transplant evaluation. At this time, the patient is undergoing preparation for a liver transplant at a quaternary care center. This consideration for the transfer was based on the patient's worsening renal function and high MELD-Na score.

## Discussion

A chylothorax typically occurs when there is damage to or obstruction of the thoracic duct [3]. The lymphatic vessels from the peritoneal cavity join together to form the thoracic duct at the posterior aspect of the abdominal aorta; at this point, the thoracic duct follows the aorta superiorly into the thoracic cavity [1]. The pathogenesis of chylothorax in liver cirrhosis is thought to be similar to that of hepatic hydrothorax [3]. High pressures in the splanchnic vessels in cirrhotic patients cause rupture of lymphatic channels; consequently, lymph is hypothesized to leak into ascitic fluid, become diluted, and migrate through the diaphragm into the lower-pressure pleural space [3]. Chylothoraces are typically exudative in nature and have been described extensively in the literature. Transudative chylothorax is an uncommon finding. In the limited literature, most cases of transudative chylothorax are attributable to nephrotic syndrome, cirrhosis, amyloidosis, and superior vena cava obstruction [4]. Romero et al. showed that in three cirrhotic patients with transudative chylothorax, 90 minutes after intraperitoneal injection of a radioisotope, the isotope was detectable in the pleural space, suggesting a transdiaphragmatic mechanism for fluid transposition [3]. In an analysis of 203 patients with chylothorax, Doerr et al. found that most instances of chylothorax in patients with liver cirrhosis are in the setting of chylous ascites [5]. Interestingly, cirrhosis is not an uncommon etiology for chylous ascites; in one study of 194 patients with chylous ascites, 11% of cases were determined to be due to cirrhosis [6]. Putting this together, the development of a transudative chylothorax in the setting of cirrhosis may be due, at least in part, to the transdiaphragmatic extravasation of chylous ascites into the pleural space. Our patient’s peritoneal fluid studies demonstrated chylous ascites, and he may have developed a transudative chylothorax by said mechanism.

The management of a chylothorax involves either conservative or surgical measures [7]. Conservative management is initially taken for a chylothorax in the setting of cirrhosis and includes drainage of fluid for symptomatic relief and measures to limit resulting nutritional deficiencies [7]. It is advised for patients to have a diet rich in low-fat medium-chain triglycerides, as these fats bypass the lymphatic system and directly enter the portal circulation [1]. These measures are necessary to reduce lymphatic flow and allow the thoracic duct to heal. Furthermore, somatostatin and octreotide can be used to decrease lymphatic flow [7]. In one study of 74 patients, Maldonado et al. found that the resolution rate of nontraumatic chylothoraces treated with periodic thoracentesis was only 19% [8]. Since lymph consists of fats, fat-soluble vitamins, and immunoglobulins, continuous drainage of the chylothorax can lead to vitamin deficiencies, malnutrition, and infection [8]. Specifically, in cirrhotic patients, who are at increased risk for variceal bleeding with bacterial translocation and spontaneous bacterial peritonitis, immunosuppression may be devastating.

If conservative treatment fails, more aggressive measures like surgery will have to be taken. Lymphangiography is a study used to identify the site of lymphatic leakage or obstruction [9]. Findings from lymphangiography can help guide surgical options and may even be therapeutic in patients with low-volume lymphatic leakage [9]. Pleurodesis is another option for patients, though it is typically a salvage treatment for refractory chylothorax in non-surgical candidates [9-10]. It aims to create adhesions between the parietal and visceral pleura to prevent the accumulation of fluid in the space [10]. Transjugular intrahepatic portosystemic shunt (TIPS) has been previously used in cirrhotic patients with recurrent chylothorax and chylous ascites [11-12]. Portal decompression with a TIPS is thought to reduce lymphatic flow and provide treatment for recurrent chylous effusions.

## Conclusions

Transudative chylothorax remains a rare entity in the current literature. The mechanism for this pleural condition is unclear, but a possible mechanism includes the transdiaphragmatic transposition of diluted lymph from the peritoneal cavity into the pleural space. The management of patients with transudative and exudative chylothorax does not differ, and strategies include conservative measures such as a low-fat diet and more aggressive interventions such as TIPS and pleurodesis. Future studies should aim to study a larger sample size of patients with transudative chylothorax to clarify the disease mechanism and identify risk factors for its development.
